# Targeted mRNA demethylation using an engineered dCas13b-ALKBH5 fusion protein

**DOI:** 10.1093/nar/gkaa269

**Published:** 2020-05-01

**Authors:** Jiexin Li, Zhuojia Chen, Feng Chen, Guoyou Xie, Yuyi Ling, Yanxi Peng, Yu Lin, Nan Luo, Cheng-Ming Chiang, Hongsheng Wang

**Affiliations:** 1 Guangdong Key Laboratory of Chiral Molecule and Drug Discovery, School of Pharmaceutical Sciences, Sun Yat-sen University, Guangzhou, Guangdong 510006, China; 2 Sun Yat-sen University Cancer Center; State Key Laboratory of Oncology in South China, Collaborative Innovation Center for Cancer Medicine, Guangzhou 510060, China; 3 Guangdong Provincial Key Laboratory of Gastroenterology, Department of Gastroenterology, Nanfang Hospital,Southern Medical University, Guangzhou, Guangdong 510006, China; 4 Simmons Comprehensive Cancer Center, Department of Pharmacology, and Department of Biochemistry, University of Texas Southwestern Medical Center, 5323 Harry Hines Boulevard, Dallas, TX 75390, USA

## Abstract

Studies on biological functions of *N*^6^-methyladenosine (m^6^A) modification in mRNA have drawn significant attention in recent years. Here we describe the construction and characterization of a CRISPR–Cas13b-based tool for targeted demethylation of specific mRNA. A fusion protein, named dm^6^ACRISPR, was created by linking a catalytically inactive Type VI-B Cas13 enzyme from *Prevotella sp. P5–125* (dPspCas13b) to m^6^A demethylase AlkB homolog 5 (ALKBH5). dm^6^ACRISPR specifically demethylates m^6^A of targeted mRNA such as cytochrome b5 form A (CYB5A) to increase its mRNA stability. It can also demethylate β-catenin-encoding CTNNB1 mRNA that contains multiple m^6^A sites to trigger its translation. In addition, the dm^6^ACRISPR system incurs efficient demethylation of targeted epitranscriptome transcripts with limited off-target effects. Targeted demethylation of transcripts coding for oncoproteins such as epidermal growth factor receptor (EGFR) and MYC can suppress proliferation of cancer cells. Together, we provide a programmable and *in vivo* manipulation tool to study mRNA modification of specific genes and their related biological functions.

## INTRODUCTION

*N* ^6^-Methyladenosine (m^6^A) is a prominent and dynamic mRNA modification that has been continuously identified in many transcripts since the 1970s (1). Thousands of RNA transcripts contain m^6^A modification with unique distribution patterns (2). m^6^A modification of mRNA is reversibly governed by methyltransferase complex (e.g. METTL3/METTL14/WTAP, referred to as ‘writers’) and demethylases (‘erasers’, FTO and/or ALKBH5) (3). Binding of ‘reader’ proteins, such as YTHDF1–3, YTHDC1/2 and IGF2BP1–3, regulates splicing, translation and decay of mRNA to affect protein production (4). Further, m^6^A modification has been reported to regulate various biological processes such as spermatogenesis (5,6), heat shock response (7), ultraviolet-induced DNA damage response (8), maternal mRNA clearance (9) and T cell homeostasis (10). Therefore epitranscriptome-based therapy has broad applications.

Current methods of manipulating RNA methylation are based primarily on modulating the expression of RNA methyltransferases or demethylases, which cause broad epigenetic changes and may activate endogenous retroviruses (11–13). Recently, flavin mononucleotide has been identified as a cell-active artificial m^6^A RNA demethylase, providing a feasible tool of using small molecules for RNA demethylation (14). However, it is difficult to study the effect of specific RNA methylation and eliminate potential side effects in therapeutic applications, due to global demethylation of transcriptome using the above methods or reagents. So far, technology for targeted manipulation of RNA methylation has rarely been reported (15). Generating an easily manipulated and targeted RNA demethylation system will have a great impact on dissecting the role of locus-specific RNA methylation in multiple biological processes.

The recently discovered CRISPR/Cas system provides an effective way to study endogenous functions and dynamic variations of nucleic acids (16–18). A nuclease-inactive DNA-targeting Cas9 (dCas9) has been used to functionally inactivate a specific gene locus (16–19). The discovery of Cas13, an RNA-targeting Cas protein, further opens the door for targeting the dynamics of endogenous RNA transcripts (20). Similar to Cas9, mutations in the nuclease domain of Cas13 can generate a catalytically dead enzyme that retains RNA-binding affinity (dCas13). A nuclease-inactivated ‘dead’ version of LwaCas13a (dLwaCas13a), when fused to EGFP, allows visualization of endogenous RNAs (21). Fusion with an A-to-I RNA-editing enzyme, such as ADAR1 or ADAR2, makes it possible for PspCas13b to edit endogenous RNA sites (22). In addition, CasRx is a programmable RNA-binding module for efficient targeting of cellular RNAs (23). All these studies indicate that a Cas13-based system can be a powerful tool to investigate the dynamics of a specific endogenous RNA event.

The dCas9 fused with epigenetic enzymes can modify epigenetic properties of the targets, including DNA methylation and histone methylation/acetylation status (16,17,24,25). A recent study showed that dCas13b-m^6^A readers can target specific transcripts of interest to regulate mRNA translation and degradation (26). In the present study, we construct and characterize a CRISPR–Cas13b-based tool to target demethylation of specific mRNA. A fusion protein was created by linking the catalytically dead Type VI-B Cas13 enzyme from *Prevotella* sp. *P5–125* (dPspCas13b) to m^6^A demethylase ALKBH5. We demonstrate that our construct combined with sgRNA, named dm^6^ACRISPR, successfully demethylates targeted mRNA in cells. We further show that the dm^6^ACRISPR system can be used to investigate the regulation of m^6^A methylation on specific endogenous mRNA. Moreover, we apply the dm^6^ACRISPR system on oncogenic targets and successfully suppress cell proliferation, suggesting that this engineered tool is instrumental for biotechnological applications. Together, this work provides a programmable and *in vivo* manipulation tool to study mRNA modification of specific genes and its potential biological functions.

## MATERIALS AND METHODS

### Cloning

The original PspCas13b plasmid (Addgene plasmid #103866), gRNA plasmid (Addgene plasmid #103854) and non-targeting gRNA plasmid (Addgene plasmid #103868) were purchased from Addgene. PspCas13b-Alkbh5, Alkbh5-PspCas13b and gRNA-containing plasmids were constructed by Synbio Technologies Company (Suzhou, China). The plasmid containing double mutations at A133H and A1058H of PspCas13b without the fusion protein was constructed as inactive Cas13b.

### Design of guide RNAs (gRNAs)

Considering that there are multiple mRNA isoforms of target genes, mRNA sequences of all isoforms were subjected to an alignment analysis, and then the common regions were used as targeting candidates for the gRNA design. gRNAs targeting 5′UTR, CDS and 3′UTR regions of target transcripts were designed and listed in Supplementary Table S1. All designed gRNAs were subjected to MEGABLAST (https://blast.ncbi.nlm.nih.gov/Blast.cgi) to avoid mismatch to unexpected mRNAs in the human genome.

### Cell culture and plasmid transfection

HEK293T (ATCC) and HeLa (ATCC) cells were cultured in Dulbecco's Modified Eagle's Medium (DMEM, Gibco/Life Technologies) supplemented with 10% fetal bovine serum (FBS, Gibco/Life Technologies), and 1% penicillin/streptomycin (P/S, Invitrogen) under 5% CO_2_. To generate *Mettl3^Mut/^^−^* HeLa cells, CRISPR-cas9 editing was used according to the published protocol (27–29). The sh-Control and sh-Mettl3 HEK293T cells were generated by lentiviral shRNA constructs. Plasmid transfection was performed with lipofectamine 3000 (Invitrogen) following manufacture's protocol. For six-well assays, cells were transfected with 1.5 μg PspCas13b-Alkbh5, Alkbh5-PspCas13b, Cas13b and 1μg gRNA for 24 h before analysis.

### SELECT qPCR

SELECT qPCR was conducted by following Xiao's protocol (30). Briefly, total RNAs were quantified by Qubit (Thermo Fisher Scientific) with Qubit™ RNA HS Assay Kit (Thermo Fisher Scientific). Total RNA (1500 ng) was mixed with 40 nM up primer, 40 nM down primer and 5 μM dNTP in 17 μl 1× CutSmart buffer (NEB). The RNA and primers were incubated at a temperature gradient: 90°C for 1 min, 80°C for 1 min, 70°C for 1 min, 60°C for 1 min, 50°C for 1 min and 40°C for 6 min. RNA and primers mixture were incubated with 3 μl of 0.01 U Bst 2.0 DNA polymerase, 0.5 U SplintR ligase and 10 nM ATP, at 40°C for 20 min, and then denatured at 80°C for 20 min. Afterward, 20 μl qPCR reaction was set up and contained 2 μl of the final reaction mixture, 200 nM SELECT primers, and 2× SYBR Green Master Mix (TaKaRa). SELECT qPCR was performed with the following program: 95°C, 5 min; 95°C, 10 s then 60°C, 35 s for 40 cycles; 95°C, 15 s; 60°C, 1 min; 95°C, 15 s; 4°C, hold. Primers for SELECT qPCR or qRT-PCR are listed in Supplementary Tables S2 and S3, respectively. C_t_ values of samples were normalized to their corresponding C_t_ values of control. All assays were performed with three independent experiments.

### m^6^A-RIP qPCR

Protein G Magnetic Beads were incubated with 1 μg m^6^A or IgG antibody in 1× reaction buffer (150 mM NaCl, 10 mM Tris–HCl, pH 7.5, 0.1% NP-40 in nuclease-free H_2_O) at 4°C for 3 h. 200 μg of extracted RNA were added into m^6^A- or IgG-conjugated Protein G Magnetic Beads at 4°C for 3 h. Bound RNAs were incubated with 100 μl Elution Buffer (75 nM NaCl, 50 nM Tris–HCl, pH 7.5, 6.25 nM EDTA, 1% (w/v) SDS, 20 mg/ml Proteinase K) for 30 min at room temperature. Eluted RNA was recovered with phenol: chloroform extraction followed by ethanol precipitation. m^6^A-RIP RNA was reverse-transcribed into cDNA and subjected to qPCR for quantification. The immunoprecipitation (IP) enrichment ratio of a transcript was calculated as the ratio of its amount in IP to that in the input yielding from the same amount of cells.

### mRNA stability

HEK293T cells were pre-transfected with gRNA and the dCas13b or dCas13b-ALKBH5 construct for 24 h and then treated with actinomycin D (Act-D, Catalog #A9415, Sigma, USA) at 5 μg/ml for the indicated time periods. Cells were collected with RNA isolated for real-time PCR. Half-life (t1/2) of mRNA was calculated using ln2/slope and including GAPDH for normalization.

### Luciferase reporter assay

Luciferase assay was conducted by using reporter lysis buffer (Catalog #E3971, Promega, U.S.A.) and luciferase assay reagents according to manufacturer's instructions. Briefly, HEK293T cells were co-transfected with pGL-5′UTR, dPspCas13b-ALKBH5, gRNA CTNNB-1 and pRL-TK (encoding Renilla Luciferase) reporter in a six-well plate. After incubation for 24 h, cells were harvested and assayed by the Dual-Glo Luciferase Assay system (Promega). Relative firefly luciferase (F-luc)/Renilla Luciferase (R-luc) was used to normalize their mRNA levels to evaluate the translation efficiency of the reporter (31).

### m^6^A sequencing (m^6^A-seq), mRNA-seq and data analysis

HEK293T cells were transfected with control gRNA or CYB5A gRNA-1 with dCas13b-ALKBH5 for 24 h. Polyadenylated RNA was isolated using TRIZOL reagent followed by isolation through FastTrack MAGMaxi mRNA isolation kit (Invitrogen). RNA fragmentation, m^6^A-seq, and library preparation were performed according to manufacturer's instructions and our previously published protocol (28). NEBNext Ultra Directional RNA Library Prep Kit (New England BioLabs, Ipswich, MA, USA) was used for library preparation. Each experiment was conducted with two biological replicates. m^6^A-seq data were analyzed according to the described protocols (31). Significant peaks with FDR < 0.05 were annotated to RefSeq database (hg19).

Total RNA was purified using the RNeasy mini kit (Qiagen, Hilden, Germany). The cDNA was generated by the use of a NuGEN Ovation RNA-Seq Systemv2 (NuGEN, San Carlos, CA, USA) was conducted according to the previous study (32). Sequencing reads were mapped to a reference human genome sequence (NCBI 36.1 [hg19] assembly by TopHat Version 2.0.6). Differentially expressed genes between conditions were statistically assessed by R/Bioconductor package edgeR (version 3.0.8). Genes with FDR <0.05 and >200 bp were called as differentially expressed. The high-throughput m^6^A and mRNA raw sequencing data have been deposited in the NCBI SRA database with accession code SRP250691.

### Statistical analysis

Data was reported as mean ± SD from at least three independent experiments unless otherwise specified. Data were analyzed by two-tailed unpaired Student's t-test between two groups and by one-way ANOVA followed by Bonferroni test for multiple comparisons. Statistical analysis was carried out using SPSS 16.0 for Windows. All statistical tests were two-sided. *P < 0.05, **P < 0.01; NS, no significant.

## RESULTS

### Design of dm^6^ACRISPR for targeted RNA demethylation

**Figure 1.**
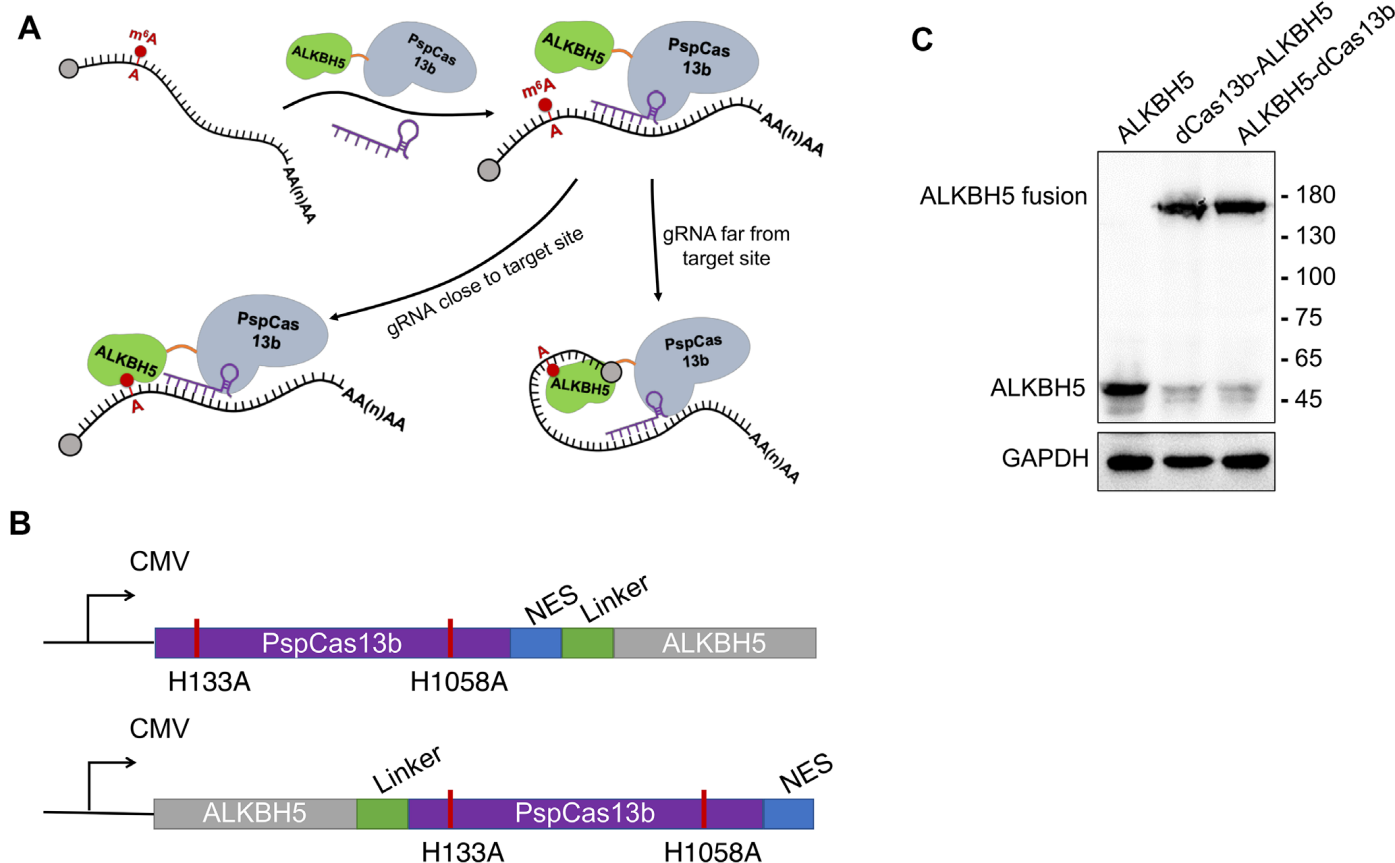
Design of dm^6^ACRISPR for targeted RNA demethylation. (**A**) Overview of site-specific RNA targeting using dCas13b-guided fusion proteins with gRNA close to or away from the target site; (**B**) Schematic representation of the domain organization of dCas13b-ALKBH5 or ALKBH5-dCas13b expression cassette; (**C**) Expression of dCas13b-ALKBH5 and ALKBH5-dCas13b fusion protein in HeLa cells measured by western blotting using ALKBH5 antibody.

To provide a simple tool to study RNA modification, we first created dCas13b-ALKBH5 fusion proteins by fusing ALKBH5 to the N- or C-terminus of inactive Cas13b (dCas13b) with a six amino-acid (GSGGGG) linker (Figure 1A and B). Concurrently, a U6 promoter-driven gRNA transcription system was cloned into the pC0043-PspCas13b gRNA backbone via the use of BbsI (22). Western blot analysis showed both dCas13b-ALKBH5 and ALKBH5-dCas13b fusions were expressed in HEK293T and HeLa cells (Figure 1C and Supplementary Figure S1A). A nuclear export signal (NES) was inserted to induce nuclear export of the fusion protein. In contrast to the nuclear localization of endogenous ALKBH5 in mammalian cells (5), dCas13b-ALKBH5 and ALKBH5-dCas13b fusions were found in both cytoplasm and nucleus, which was confirmed by immunofluorescence (Supplementary Figure S1B) and Western blot analysis (Supplementary Figure S1C).

### dm^6^ACRISPR induces demethylation of single m^6^A-modified mRNA

**Figure 2.**
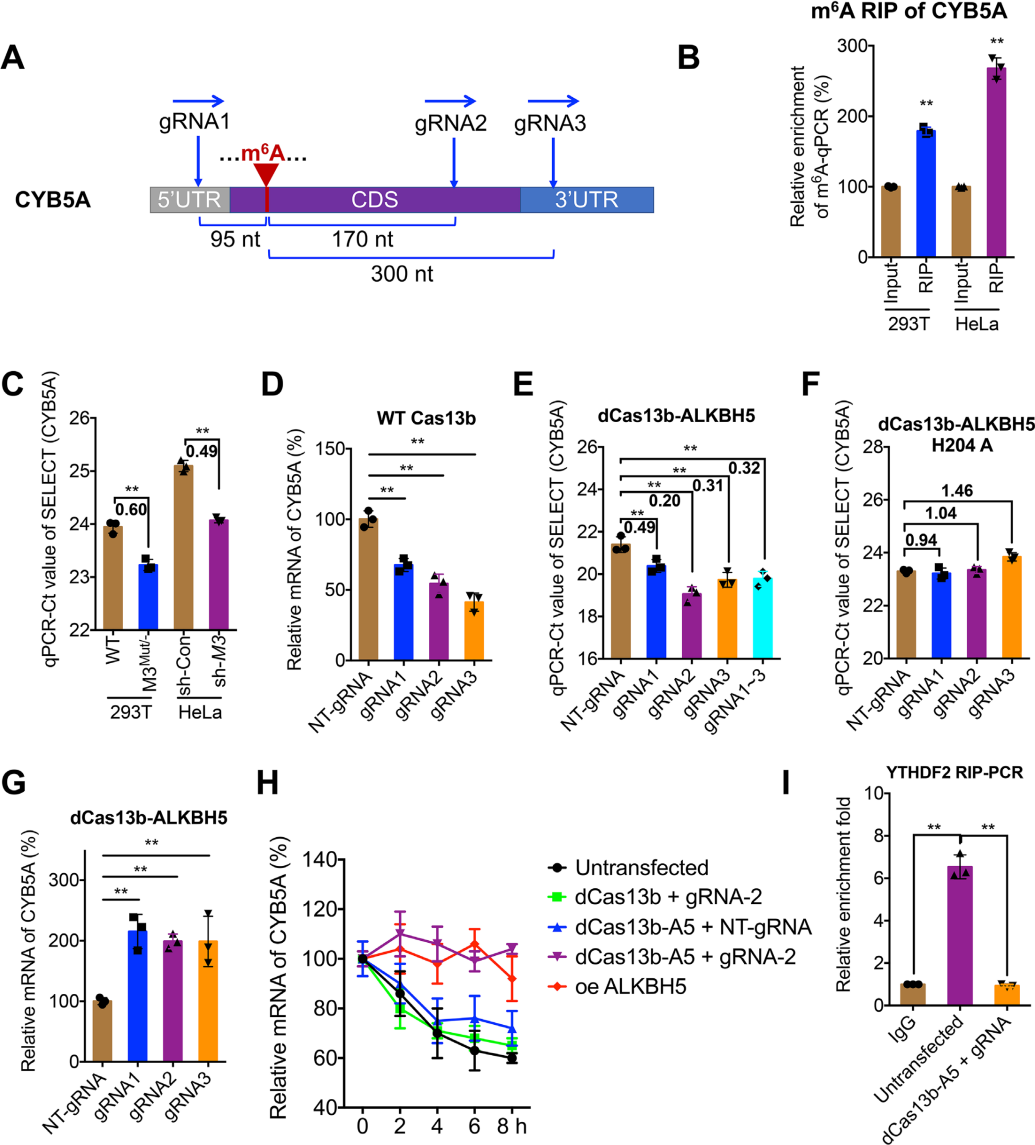
dm^6^ACRISPR induces demethylation of single m^6^A-modified mRNA. (**A**) Schematic representation of positions of the m^6^A site within CYB5A mRNA and regions targeted by three gRNAs; (**B**) m^6^A enrichment of CYB5A mRNA in HeLa and HEK293T cells was analyzed by m^6^A RIP-qPCR analysis using m^6^A antibody; (**C**) Threshold cycle (Ct) of qPCR showing SELECT results for detecting the m^6^A site in CYB5A at A48 in HeLa (wide-type) and HEK293T cells (sh-Control) compared to their corresponding METTL3-knockdown cells (M3KD) with fold changes listed; (**D**) mRNA expression of CYB5A in HEK293T cells cotransfected with wild-type (WT) Cas13b and NT-gRNA (control) or gRNA1/2/3 for 24 h; (**E, F**) Threshold cycle (Ct) of SELECT-qPCR detecting the m^6^A site in CYB5A at A48 in HEK293T cells transfected with dCas13b-ALKBH5 (E) or dCas13b-ALKBH5 H204A (F) combined with NT-gRNA or gRNA1/2/3, respectively, for 24 h, with fold changes listed; (**G**) mRNA levels of CYB5A in HEK293T cells transfected with dCas13b-ALKBH5 combined with NT-gRNA or gRNA1/2/3, respectively, for 24 h; (**H**) HEK293T cells were pre-transfected as indicated for 24 h and then further treated with Act-D for the indicated times. The mRNA levels of CYB5A were measured by qRT-PCR. oe, overexpression; (**I**) HEK293T cells were pre-transfected as indicated for 24 h. Binding between YTHDF2 and CYB5A mRNAs was checked by RIP-qPCR using YTHDF2 antibody. Data are presented as mean ± SD from three independent experiments. **P < 0.01 by Student's *t* test (B and C) or one-way ANOVA (D, E, F, G, H and I).

To test the utility of dm^6^ACRISPR for targeted m^6^A demethylation, we selected potential candidate transcripts according to the following criteria: (i) the FPKM (fragments per kilobase of transcript per million fragments mapped) of a transcript is >300 in m^6^A-seq according to our previous study (28); and (ii) only one m^6^A peak site was identified for the transcript. Finally, cytochrome b5 form A (CYB5A) was chosen to investigate the demethylation effect of dm^6^ACRISPR (Figure 2A). m^6^A-RIP-seq data showed a unique m^6^A peak in the CYB5A CDS region at the A48 residue (Supplementary Figure S2A). The m^6^A modification in CYB5A mRNA was confirmed by m^6^A-RIP-qPCR in METTL3-knockdown HeLa and HEK293T and their corresponding control cells (Figure 2B and Supplementary Figure S2B). Further, the m^6^A site in the CDS region of CYB5A at A48 was verified by a single-base elongation- and ligation-based qPCR amplification method (termed ‘SELECT’) (30) (Figure 2C), while the nearby nucleotide GAC (A17) showed no m^6^A modification (Supplementary Figure S2C). Our data confirmed that the m^6^A at A48 of the CYB5A CDS exists and is reversibly modified.

The mRNA of CYB5A was targeted by three gRNAs at distinct positions, which are in the conserved region among the three CYB5A isoforms, around the m^6^A site (Figure 2A). To test the efficiency of gRNAs, we analyzed the mRNA levels of CYB5A in cells transfected with gRNAs and wild-type Cas13b, which cleaves targeted mRNA. Our data showed that all three gRNAs combined with wild-type Cas13b significantly decreased the mRNA levels of CYB5A (Figure 2D), suggesting that all three gRNAs can efficiently recognize CYB5A. In contrast, transfection of gRNAs alone (Supplementary Figure S2D) or gRNAs combined with dCas13b (Supplementary Figure S2E) had no effect on mRNA levels of CYB5A.

To minimize the off-target effect of overexpression artifacts (33), we optimized the transfection amount of plasmids. The results showed upregulation of targets was induced by dCas13b-ALKBH5 when 6 μg of dCas13b-ALKBH5 plasmid per 1 × 10^6^ cells were used (Supplementary Figure S2F), implying that an off-target effect exists under high doses of dCas13b-ALKBH5. Therefore, the dose of 1.5 μg dCas13b-ALKBH5 plasmid per 1 × 10^6^ cells was used for further studies to reduce the off-target effect.

We then verified the effect of dm^6^ACRISPR (gRNAs for CYB5A and dCas13b-ALKBH5) on m^6^A modification of CYB5A. SELECT showed that all three gRNAs combined with dCas13b-ALKBH5 significantly decreased the m^6^A levels of the targeted site (Figure 2E). The strongest demethylation on the CYB5A CDS was observed with gRNA2, which targets a ∼170 nt downstream region from the m^6^A site and resulted in 80.2 ± 3.6% demethylation (2-ΔCt method). The dCas13b-ALKBH5-mediated demethylation of CYB5A was further confirmed by m^6^A-RIP-qPCR (Supplementary Figure S2G). When cells were co-transfected with gRNAs and dCas13b-ALKBH5 H204A, which is a catalytically inactive mutant of ALKBH5 (34), there was no demethylation on CYB5A mRNA (Figure 2F). Intriguingly, gRNAs and dCas13b-ALKBH5 transfection led to significant upregulation of CYB5A mRNA (Figure 2G). To investigate whether dCas13b-ALKBH5-induced upregulation of CYB5A was due to m^6^A-mediated mRNA decay, we measured CYB5A mRNA half-life. Results showed that both targeted demethylation by dCas13b-ALKBH5/gRNA-2 or global demethylation by ALKBH5 could significantly stabilize CYB5A mRNA, while dCas13b with gRNA-2 or dCas13b-ALKBH5 with NT-gRNA had no effect on mRNA stability (Figure 2H). This might be due to dm^6^ACRISPR-decreased binding between CYB5A mRNA and YTHDF2 (Figure 2I), which mediates mRNA decay through P-body and other forms (35). Results showed that induction on CYB5A mRNA levels of dCas13b-ALKBH5 was significantly greater than that of ALKBH5-dCas13b (Supplementary Figure S2H), thus dCas13b-ALKBH5 was used for most of the remaining studies. Collectively, these results suggest that dm^6^ACRISPR can increase mRNA stability via demethylating m^6^A at the CDS in the case of CYB5A.

### dm^6^ACRISPR induces demethylation of multiple m^6^A sites of 5′UTR

**Figure 3.**
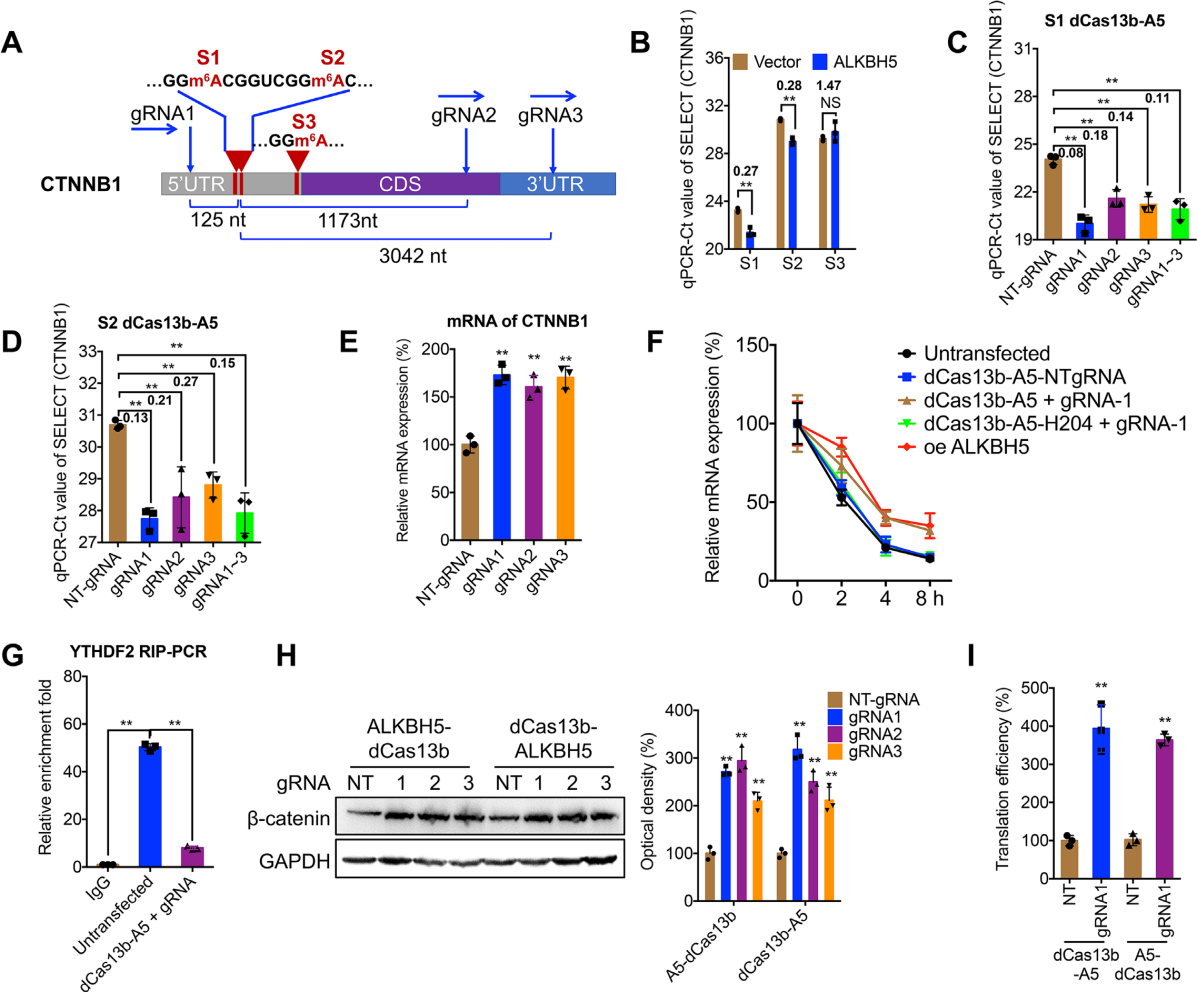
dm^6^ACRISPR induces demethylation of multiple m^6^A sites of 5′UTR. (**A**) Schematic representation of positions of m^6^A sites within CTNNB1 mRNA and regions targeted by three gRNAs; (**B**) Threshold cycle (Ct) of qPCR showing SELECT results for detecting m^6^A in three potential m^6^A sites of CTNNB1 in HEK293T cells transfected with vector control (pcDNA3.1) or pcDNA/ALKBH5, with fold changes listed; (**C**, **D**) Threshold cycle (Ct) of qPCR showing SELECT results for detecting the m^6^A S1 (C) or S2 (D) site in CTNNB1 in HEK293T cells transfected with dCas13b-ALKBH5 combined with NT-gRNA or gRNA1/2/3, respectively, for 24 h, with fold changes listed; (**E**) mRNA levels of CTNNB1 in HEK293T cells transfected with dCas13b-ALKBH5 combined with NT-gRNA or gRNA1/2/3, respectively, for 24 h; (**F**) HEK293T cells were pre-transfected as indicated for 24 h and then further treated with Act-D for the indicated time periods. The mRNA levels of CTNNB1 were measured by qRT-PCR. oe, overexpression; (**G**) HEK293T cells were pre-transfected as indicated for 24 h. Binding between YTHDF2 and CTNNB1 mRNA was checked by RIP-qPCR using YTHDF2 antibody; (**H**) Protein expression of β-catenin in HEK293T cells transfected with dCas13b-ALKBH5 combined with NT-gRNA (NT) or gRNA1/2/3, respectively, for 24 h was checked by western blot analysis (*left*) and quantitatively analyzed (*right*); (**I**) cells were transfected with pGL-5′UTR, NT-gRNA (NT) or gRNA1, and dCas13b-ALKBH5 or ALKBH5-dCas13b and pRL-TK reporter for 24 h. Translation efficiency is defined as the quotient of reporter protein production (F-luc/R-luc) divided by mRNA abundance (31). Data are presented as mean ± SD from three independent experiments. ** *P* < 0.01 by Student's *t* test (B and I) or one-way ANOVA (C, D, E, F, G and H).

To test the capability of dm^6^ACRISPR on a transcript with multiple m^6^A sites, CTNNB1 (the gene encoding β-catenin) was chosen for further investigation. Results from m^6^A-RIP-seq indicated that there might be 3 potential m^6^A sites at 5′UTR: position A180 (Site 1, S1), A188 (S2) and A266 (S3) (Figures 3A and Supplementary Figure S3A). m^6^A-RIP-PCR confirmed that CTNNB1 was m^6^A-modified in both HEK293T and HeLa cells (Supplementary Figure S3B). The mRNA of CTNNB1 was targeted by three gRNAs, which bind at <150 nt upstream or distal (3042 nt) to the m^6^A sites, respectively (Figure 3A).

qRT-PCR showed that transfection of these three gRNAs with wild-type Cas13b significantly decreased the expression of CTNNB1 mRNA (Supplementary Figure S3C), suggesting that all gRNAs worked efficiently in cells. SELECT showed that only S1 and S2 m^6^A sites were reversible in cells transiently overexpressing ALKBH5 (Figure 3B). The gRNA1, which targets a site located at 125 nt upstream of the m^6^A S1 site, resulted in 91.5 ± 4.2% and 87.1 ± 2.6% average demethylation (2^−ΔCt^ method) in S1 (Figure 3C) and S2 (Figure 3D) sites, respectively. However, there is no significant change of m^6^A level in the S3 site using either ALKBH5 overexpression (Figure 3B) or dm^6^ACRISPR (Supplementary Figure S3D). Furthermore, overexpression of dCas13b-ALKBH5 H204A did not demethylate CTNNB1 mRNA at either the S1 (Supplementary Figure S3E) or S2 (Supplementary Figure S3F) site, confirming that demethylation of CTNNB1 mRNA was achieved by dm^6^ACRISPR specifically.

Similar to CYB5A, dm^6^ACRISPR increased mRNA levels of CTNNB1 (Figure 3E). However, dCas13b-ALKBH5 H204A had no effect on the mRNA levels of CTNNB1 (Supplementary Figure S3G). RNA stability assay showed that dm^6^ACRISPR increased the mRNA half-life of CTNNB1 (Figure 3F), suggesting that demethylation of 5′UTR of CTNNB1 also increased the stability of mRNA. Consistently, either dCas13b-ALKBH5 with NT-gRNA or dCas13b-ALKBH5 H204A could influence the mRNA stability of CTNNB1 (Figure 3F). This might be due to dm^6^ACRISPR-decreased binding between CTNNB1 mRNA and YTHDF2 (Figure 3G). Further, protein levels of β-catenin were obviously increased after CRISPR treatment (Figure 3H). Considering that the elevated levels of protein (about 3-fold) were much greater than that of mRNA (less than 2-fold), our data indicated that m^6^A on CTNNB1 5′UTR may also regulate its translation. To verify this, we subcloned the 5′UTR region prior to the F-Luc coding region in pGL3-Basic to generate a translational reporter (Supplementary Figure S3H). Both dCas13b-ALKBH5 and ALKBH5-dCas13b combined with gRNA1 significantly increased the translation efficiency of F-Luc than that of control NT-gRNA (Figure 3I). Together, these results not only verified demethylation of dm^6^ACRISPR on a transcript with multiple m^6^A sites, but also suggested that 5′UTR methylation could regulate both mRNA stability and translation of CTNNB1.

### Specificity and non-additive effects of dm^6^ACRISPR on m^6^A demethylation

**Figure 4.**
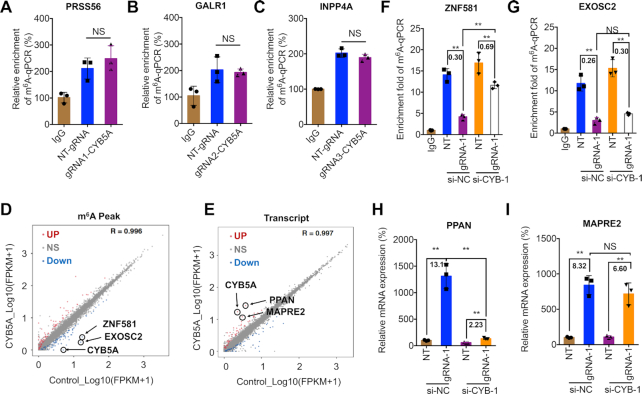
Specificity and non-additive effects of dm^6^ACRISPR on m^6^A demethylation. (**A–C**) Cells were transfected with dCas13b-ALKBH5 and NT-gRNA or gRNA1/2/3 of CYB5A, respectively, for 24 h, with m^6^A levels of PRSS56 (A), GALR1 (B), or INPP4A (C) measured by m^6^A -RIP-qPCR analysis; (**D**) Smooth scatter plot displaying mean methylation in NT-gRNA (control) or CYB5A gRNA-1-transfected HEK293T cells for 24 h with dCas13b-ALKBH5 (total 25 888 peaks). Red dots indicate peaks with significant upregulation while blue dots indicate peaks with significant downregulation in methylation (*P* < 0.05); (**E**) Smooth scatter plot displaying mean expression of transcripts in NT-gRNA (control) or CYB5A gRNA-1-transfected HEK293T cells for 24 h with dCas13b-ALKBH5 (total 25,603 transcripts). Red dots indicate transcripts with significant upregulation while blue dots indicate transcripts with significant downregulation (*P* < 0.05); (**F**, **G**) HEK293T cells were transfected with si-NC or si-CYB5A-1 together with NT-gRNA (NT) or CYB5A gRNA-1 combined with dCas13b-ALKBH5 for 24 h. The m^6^A fold enrichment of ZNF581 (F) or EXOSC2 (G) was analyzed by m^6^A RIP-qPCR analysis, with fold changes listed; (**H**, **I**) cells were transfected with si-NC or si-CYB5A-1 together with NT-gRNA (NT) or CYB5A gRNA-1 for 24 h. Relative mRNA levels of PPAN (H) or MAPRE2 (I) were analyzed by qPCR analysis, with fold changes listed. Data are presented as mean ± SD from three independent experiments. ** *P* < 0.01, NS, no significant, by one-way ANOVA with Bonferroni test.

Having shown efficient demethylation of the targeted sites, a question was raised if off-target RNA demethylation could be introduced at additional transcriptome locations. To examine this, we predicted off-target gRNA binding sites for CYB5A gRNAs by BLASTN using ‘somewhat similar sequences’. PRSS56, GALR1 and INPP4A had the highest matches for gRNA1/2/3 of CYB5A, with a match of 86%, 92% and 77%, respectively (Supplementary Figure S4A). The methylation status of these off-target sites was measured after transfection with dCas13b-ALKBH5 and their corresponding gRNAs. The m^6^A-RIP-qPCR results showed no significant effect on methylation levels of PRSS56, GALR1 or INPP4A (Figure 4A–C), although two of them had slightly increased demethylation (4.8% and 6.4% decrease of methylation on GALR1 and INPP4A, respectively). Compared to the 80.2% and 69.4% demethylation efficiency on CYB5A mRNA using gRNA2 and gRNA3, the off-target effect on tested transcripts was limited. No off-target transcript was observed for gRNAs of CTNNB1 (matches < 50%). We further evaluated the potential effect of gRNA2-CYB5A on CTNNB1 and gRNA1-CTNNB1 on CYB5A. Our data showed that gRNA1-CTNNB1 together with dCas13b-ALKBH5 had no effect on mRNA stability of CYB5A (Supplementary Figure S4B). Consistently, gRNA2-CYB5A together with dCas13b-ALKBH5 had no effect on mRNA stability of CTNNB1 (Supplementary Figure S4C).

To exclude the possibility that our system alters global demethylation of transcriptome (36), we performed m^6^A sequencing (m^6^A-seq) to map m^6^A methylomes in cells transfected with NT-gRNA or gRNA-1 for CYB5A. The mRNA-seq was performed to evaluate potential off-target effects of dm^6^ACRISPR on transcriptome. Among 25 888 measured m^6^A peaks in the transcriptome (Supplementary Table S4), there were 78 (0.30%) down-regulated and 76 (0.29%) up-regulated m^6^A peaks with a significant change (*P* < 0.05) in gRNA-1 for the CYB5A group when compared with that in the NT-gRNA group (Figure 4D). Further, among the 26303 identified transcripts (Supplementary Table S5), there were 48 (0.18%) down-regulated and 51 (0.19%) up-regulated transcripts with a significant change (*P* < 0.05) in the CYB5A group when compared with that in the NT-gRNA group (Figure 4E). Further, GO (Supplementary Figure S4D) and KEGG (Supplementary Figure S4E) analysis showed that variated m^6^A peaks with a significant change (*P* < 0.05) induced by gRNA-1 for the CYB5A group may be related to RNA polymerase II holoenzyme and cell adhesion molecules, respectively. As to the variated mRNA induced by gRNA-1 for CYB5A, GO (Supplementary Figure S4F) and KEGG (Supplementary Figure S4G) analysis showed that they are related to nucleic acid binding and TGF-β signaling pathways, respectively. However, the number of peaks and genes involved in the variated clusters was very few, which confirmed that the off-target effect of dm^6^ACRISPR/CYB5A was weak.

In order to evaluate the methylation variation directly or indirectly mediated by dm^6^ACRISPR/CYB5A, we knocked down the expression of CYB5A via siRNA (Supplementary Figure S4H and S4I). Our data showed that si-CYB5A attenuated dm^6^ACRISPR-induced demethylation of ZNF581 (Figure 4F), while had no effect on dm^6^ACRISPR-induced demethylation of EXOSC2 (Figure 4G), suggesting that CYB5A was involved in dm^6^ACRISPR-induced demethylation of ZNF581, but not of EXOSC2. Further, si-CYB5A could attenuate dm^6^ACRISPR-induced upregulation of PPAN (Figure 4H) but had no effect on that of MAPRE2 (Figure 4I). Collectively, all these data suggest that dm^6^ACRISPR has off-targets on methylation of certain transcripts such as EXOSC2, but the effect is limited to <0.2% transcripts in the transcriptome.

Subsequently, we tested whether co-targeting the same loci with multiple gRNAs could enhance the demethylation levels. The results showed targeting all three gRNAs to the CYB5A CDS did not increase the demethylation efficiency (68.3 ± 3.5%, Figure 2E) when compared to a single gRNA-targeting experiment (e.g. gRNA2, 80.2 ± 3.6%). Consistently, targeting all three gRNAs to CTNNB1 5′UTR also did not result in increased methylation efficiency (89.1 ± 3.6%, Figure 3C) when compared to that of gRNA1 of CTNNB1 (91.5 ± 4.2%) for the S1 site, suggesting that there is a non-additive effect of dm^6^ACRISPR system on RNA demethylation.

### Targeting m^6^A of oncogene transcripts by dm^6^ACRISPR regulates cell proliferation

**Figure 5.**
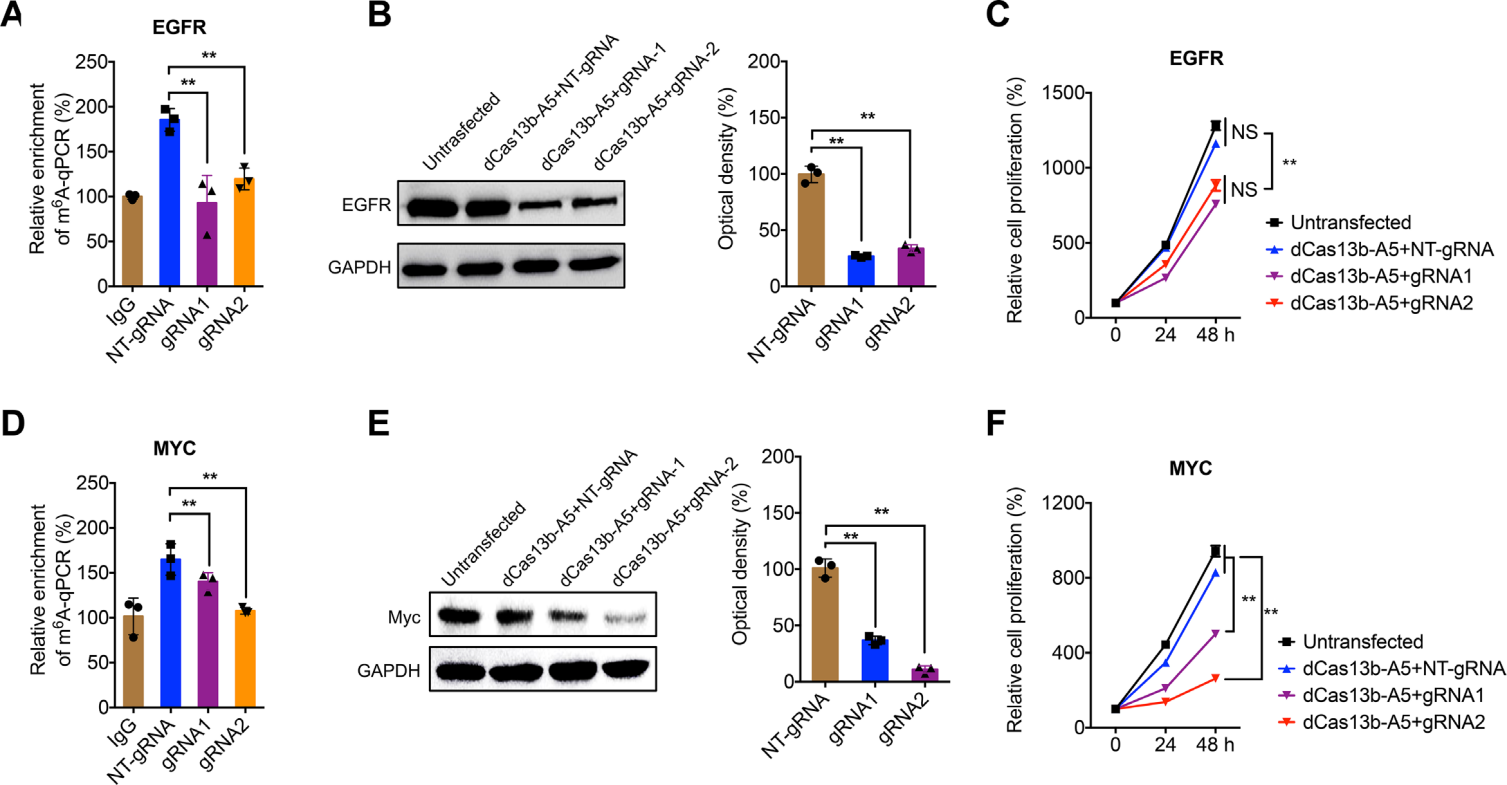
Targeting m^6^A of oncogene transcripts by dm^6^ACRISPR regulates cell proliferation. (**A**) m^6^A RIP-qPCR analysis of EGFR mRNA in HeLa cells transfected with dCas13b-ALKBH5 combined with NT-gRNA or gRNA1/2, respectively, for 24 h; (**B**) Protein expression of EGFR in HeLa cells untransfected or transfected with dCas13b-ALKBH5 combined with NT-gRNA or gRNA1/2, respectively, for 24 h, and checked by western blot analysis (*left*) and quantitatively analyzed (*right*); (**C**) Proliferation of HeLa cells untransfected or transfected with dCas13b-ALKBH5 combined with NT-gRNA or gRNA1/2, respectively, for the indicated time periods; (**D**) m^6^A RIP-qPCR analysis of MYC mRNA in HeLa cells transfected with dCas13b-ALKBH5 combined with NT-gRNA or gRNA1/2, respectively, for 24 h; (**E**) Protein expression of MYC in HeLa cells untransfected or transfected with dCas13b-ALKBH5 combined with NT-gRNA or gRNA1/2, respectively, for 24 h, and checked by western blot analysis (*left*) and quantitatively analyzed (*right*); (**F**) Proliferation of HeLa cells untransfected or transfected with dCas13b-ALKBH5 combined with NT-gRNA or gRNA1/2, respectively, for the indicated time periods. Data are presented as mean ± SD from three independent experiments. ** *P* < 0.01, NS, no significant, by one-way ANOVA with Bonferroni test.

To explore potential applications of dm^6^ACRISPR, we designed gRNAs targeting m^6^A modification of EGFR and MYC (Supplementary Figure S5A-S5C), two well-studied oncogenes which can be m^6^A modified (37) and positively regulate cell proliferation (38). Two gRNAs targeting the conserved regions among isoforms of EGFR and MYC, respectively, were designed (Supplementary Figure S5C). Our data showed that dCas13b-ALKBH5 with gRNAs significantly decreased m^6^A enrichment of EGFR (Figure 5A). Further, dCas13b-ALKBH5 with gRNAs decreased the protein expression of EGFR (Figure 5B). Cell proliferation assay revealed that dm^6^ACRISPR targeting EGFR decreased the proliferation of HeLa cells (Figure 5C). Consistently, dm^6^ACRISPR targeting MYC induced demethylation of MYC mRNA (Figure 5D), suppressed its protein expression (Figure 5E) and decreased cell proliferation (Figure 5F). Further, dm^6^ACRISPR targeting EGFR and MYC decreased their mRNA levels, respectively (Supplementary Figure S5D and E). Since the decreased levels of mRNA were less than that of protein (Figure 5B and E, and Supplementary Figure S5D and E), dm^6^ACRISPR may also regulate the translation of EGFR and MYC, consistent with YTHDF1-elevated mRNA translation (31). RIP-qPCR further revealed that dm^6^ACRISPR decreased the binding between EGFR mRNA and YTHDF1 (Supplementary Figure S5F) and the binding between MYC mRNA and YTHDF1 (Supplementary Figure S5G). These results confirmed that targeted demethylation of functional gene transcripts can regulate their cellular activities.

## DISCUSSION

Through CRISPR/Cas engineering, we found that a single gRNA co-transfected with dCas13b-ALKBH5, named dm^6^ACRISPR, could cause robust mRNA demethylation for a particular transcript with limited off-target effects. Specifically, dm^6^ACRISPR can lead to the demethylation of a single m^6^A site in the CYB5A CDS and multiple m^6^A sites in the CTNNB1 5′UTR. Our system incurs efficient demethylation of targeted transcripts with limited off-target effects on epitranscriptome. Targeted demethylation of oncogene transcripts can decrease their expression, resulting in suppression of cell proliferation. Together, we provide a programmable and *in vivo* manipulation tool to targeted demethylation of specific mRNA in transcriptome.

Unlike gRNAs for the Cas9 system that requires a protospacer adjacent motif (PAM) at the editing site (39), gRNAs for Cas13 have no targeting sequence constraints such as PAM and no motif preference surrounding the target site (22). Also, the high mismatch intolerance of gRNA gives the dm^6^ACRISPR system more advantages compared with the other nucleic acid-editing tools (22). Strikingly, RNA demethylation seems not to be influenced by either the 5′ or 3′ sequence of the dCas13b-targeted site, but it may be dependent on space between dCas13b-targeted and m^6^A-methylated sites. Our data indicated that spacing between 100∼300 nt might enhance the demethylation efficiency. Consistently, peaks of methylation ∼200 nt both upstream and downstream of the PAM site have also been observed in DNA methylation using dCas9–Dnmt3a–Dnmt3L methyltransferase (40), zinc fingers (41) and TALE (42) effectors. Our results revealed that methylation up to 3000 nt away from the target site could also be demethylated, similar to the DNA demethylation system using dCas9–peptide repeats and the scFv–TET1 catalytic domain (25) or dCas9-MQ1 (43) fusions. Further, a non-additive effect of the dm^6^ACRISPR system was observed. It may be caused (i) by less co-transfection of multiple gRNAs, (ii) by competition of gRNAs for dCas13b-ALKBH5 protein or (iii) by steric hindrance when multiple dCas13b-ALKBH5 proteins are bound on a single transcript, thus preventing an increase of demethylation efficiency. Our finding was reminiscent of previous reports using dCas9-fused p300 to activate gene expression (16) or dCas9/sgRNA2.0-directed demethylation for DNA methylation (44).

Theoretically, the most efficient RNA demethylation should be deposited near the dCas13b-binding sites. We observed efficient RNA demethylation for CYB5A by targeting >100 nt away from the methylation site. There were very limited demethylation effects at two of the predicted off-target regions (GALR1 and INPP4A, respectively), which was much weaker than the demethylation at corresponding on-target sites by the same gRNAs. m^6^A-seq suggests that dm^6^ACRISPR may have limited off-target effects on epitranscriptome with 78 (0.30%) down-regulated and 76 (0.29%) up-regulated m^6^A peaks beside the target sites. It might be due to transient dCas13b binding or touching with highly methylated transcripts, leading to non-specific demethylation. Similar off-target effects have been observed in DNA methylation using dCas9–Dnmt3a–Dnmt3L methyltransferase (40). Off-targets could be potentially reduced by decreasing the level of dCas13b fusion protein and developing specific Cas13b mutants. Intriguingly, our data also indicate that CYB5A may influence m^6^A methylation of other transcripts such as ZNF581, likely due to CYB5A influencing the expression of RNA-binding proteins to alter RNA secondary structure or recruit methyltransferase/demethylase.

Our epigenetic editing tools targeting RNA demethylation caused downregulation of targeted gene expression (EGFR and MYC) and suppression of cell proliferation, indicating that dm^6^ACRISPR could be used as a universal tool for gene repression and regulation of cellular functions. However, demethylation of CYB5A and CTNNB1 increased target transcript expression via upregulation of mRNA stability. The diverse effects of dm^6^ACRISPR were mediated by different ‘reader’ proteins (4). RNA modification-based therapies could achieve changes in genetic information with the advantage of being transient, which eliminates the concern of introducing permanent alterations via targeting DNA. Another potential advantage of targeted demethylation is that it is possible to trigger durable effects on specific targets. dm^6^ACRISPR can significantly demethylate CYB5A after transfection for 72 h (data not shown).

Our future work includes modification and optimization of the dm^6^ACRISPR system and further explores potential applications, especially establishing the functional significance of RNA demethylation in cellular function. Considering that CRISPR-based manipulation of the epigenome has achieved great success in applications, our newly developed dm^6^ACRISPR targeting RNA demethylation has unique potential to correct epimutations in disease states.

## DATA AVAILABILITY

Data supporting the findings of this study are available within the paper and its Supplementary Data files. The high-throughput m^6^A and mRNA raw sequencing data have been deposited in the NCBI SRA database with accession code SRP250691.

## Supplementary Material

gkaa269_Supplemental_Files
