# Supplementary material for: Targeted mRNA demethylation using an engineered dCas13b-ALKBH5 fusion protein
Source: Nucleic Acids Res. 2020 May 1;48(10):5684–94. doi: 10.1093/nar/gkaa269 (PMC7261189; doi:10.1093/nar/gkaa269)
Supplement: gkaa269_Supplemental_Files [file gkaa269_supplemental_files.zip › SI_2nd revision.pdf]

Supplementary data for

Targeted mRNA demethylation using an engineered dCas13b-ALKBH5 fusion protein

Li et al.

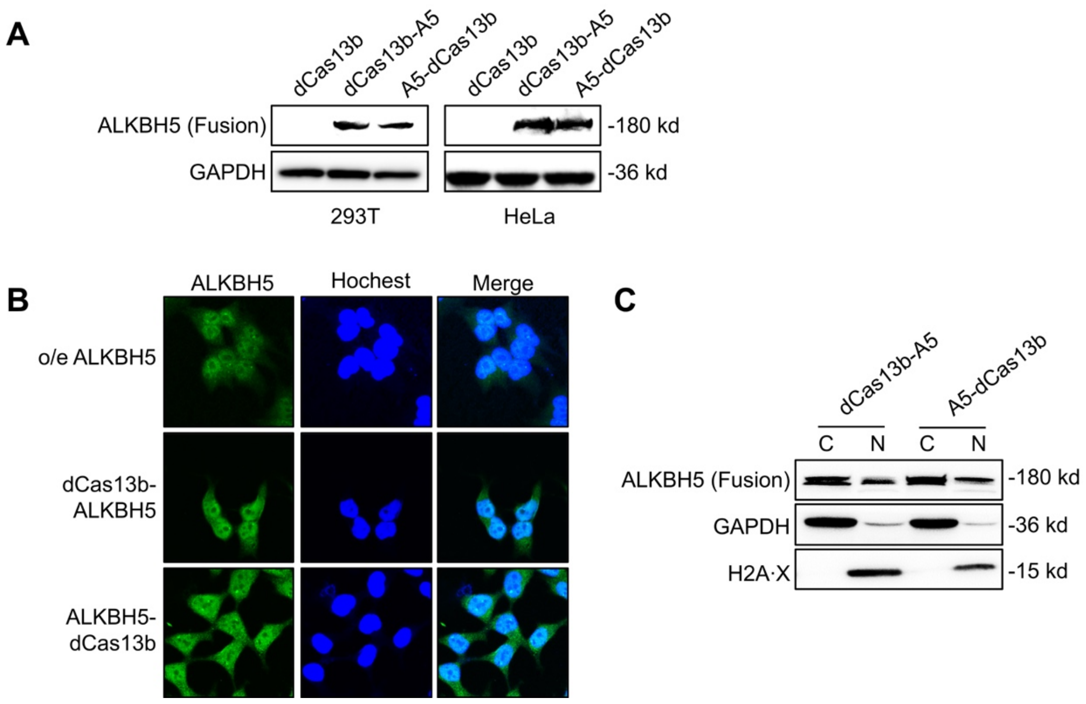

Figure S1. Design of dm^{6}ACRISPR for targeted RNA demethylation.

(A) Expression of fusion protein dCas13b-ALKBH5 or ALKBH5-dCas13b in transfected cells was measured by Western blot analysis with anti-ALKBH5 as the primary antibody;

(B) Subcellular localization of the fusion protein in HeLa cells after transfection with the ALKBH5, dCas13b-ALKBH5, or ALKBH5-dCas13b construct for 24 h checked by confocal imaging using antibody against ALKBH5. o/e, over expression;

(C) Subcellular localization of the fusion protein in HeLa cells after transfection with dCas13b-ALKBH5 or ALKBH5-dCas13b for 24 h checked by Western blot analysis. C, cytoplasm; N, nucleus.

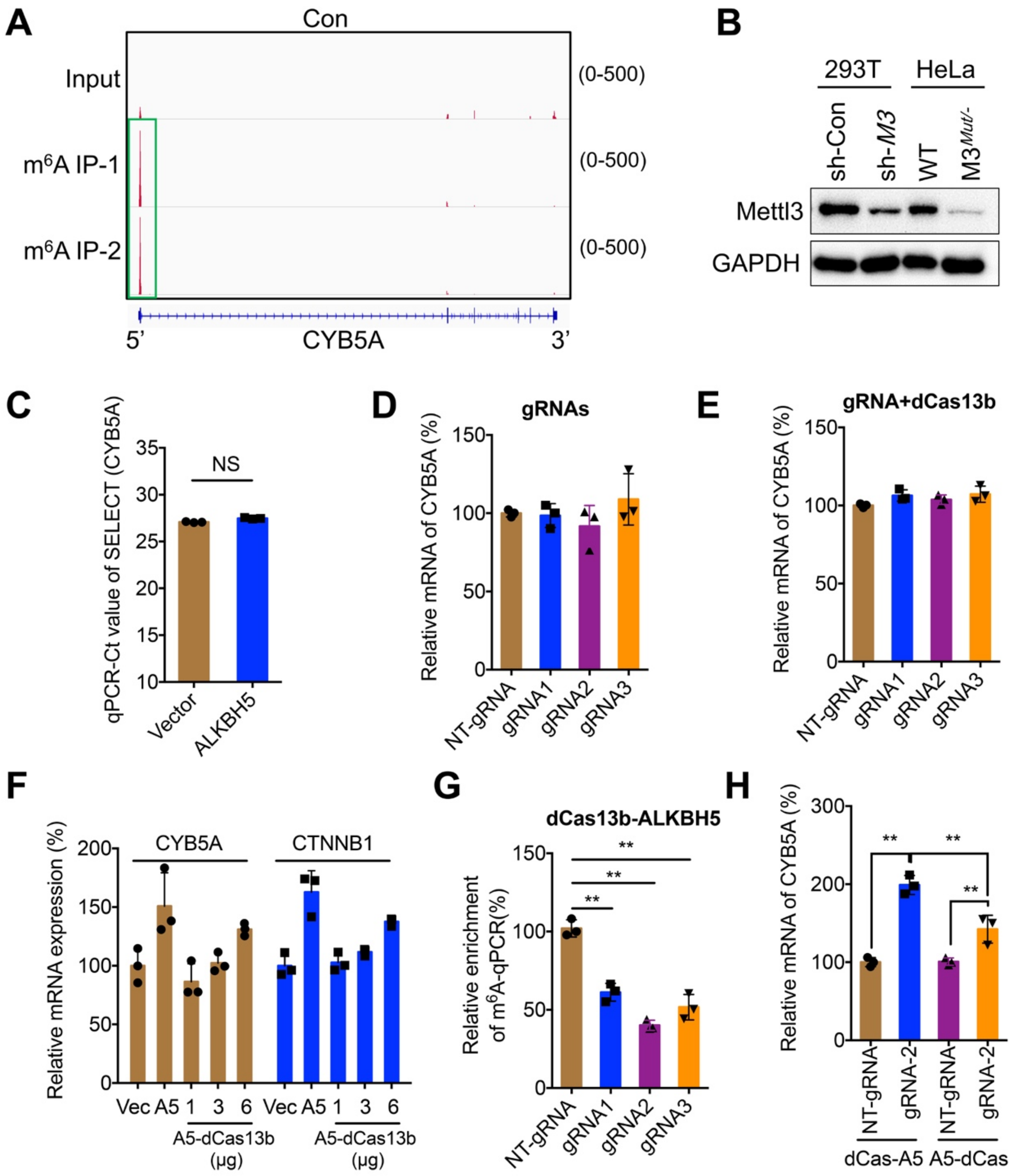

Figure S2. dm^{6}ACRISPR-induced m^{6}A demethylation at CDS increases mRNA stability.

(A) m^{6}A peaks were enriched in the CDS of CYB5A genes from m^{6}A RIP-seq data. Squares mark m^{6}A peaks in HeLa cells;

(B) Protein levels of Mettl3 in METTL3-knockdown HeLa and HEK293T and their corresponding control cells;

(C) Threshold cycle (Ct) of qPCR showing SELECT results for detecting the potential m^{6}A site in the CDS region of CYB5A at A17 in HeLa cells transfected with pcDNA (vector) or pcDNA/ALKBH5 for 24 h;

(D) mRNA levels of CYB5A in HEK293T cells transfected with NT-gRNA or individual gRNAs for 24 h;

(E) mRNA levels of CYB5A in HEK293T cells transfected with dCas13b and NT-gRNA or individual gRNAs for 24 h;

(F) Cells ($1x10^6$) were transfected with vector control (dCas13b), pcDNA/ALKBH5 construct (1μg), or increasing amounts of dCas13b-ALKBH5 for 24 h. The mRNA levels of CYB5A and CTNNB1 were checked by qRT-PCR;

(G) m^6A RIP-qPCR analysis of CYB5A mRNA in HEK293T cells transfected with dCas13b-ALKBH5 combined with NT-gRNA or gRNA1/2/3, respectively, for 24 h;

(H) The mRNA levels of CYB5A in HEK293T cells transfected with dCas13b-ALKBH5 or ALKBH5-dCas13b combined with NT-gRNA or gRNA-2, respectively, for 24 h.

Data are presented as mean ± SD from three independent experiments. ** $p < 0.01$ by One-Way ANOVA with Bonferroni test. NS, no significant.

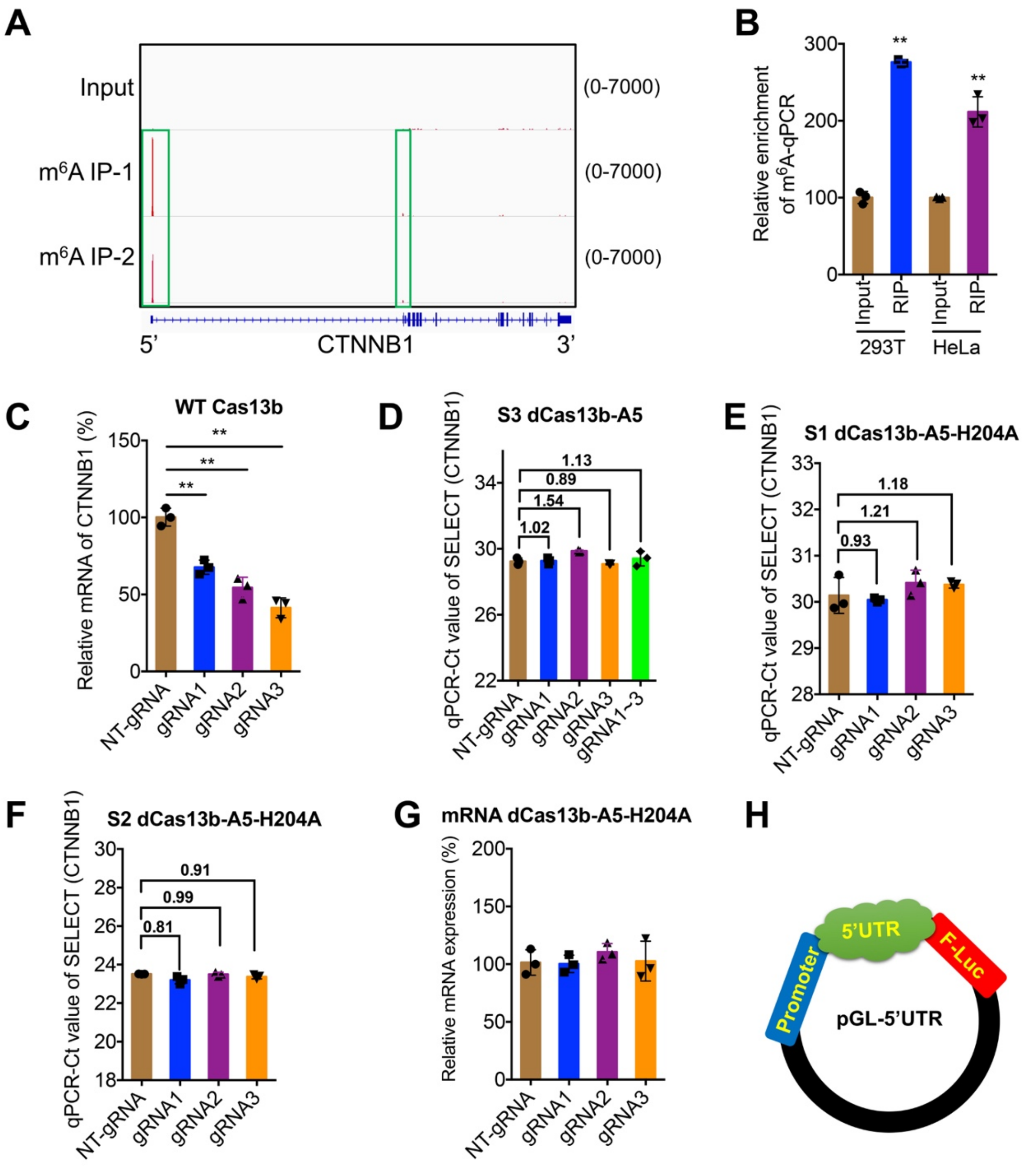

Figure S3. dm^{6}ACRISPR induces demethylation of multiple m^{6}A sites in CTNNB1 5'UTR.

(A) m^{6}A peaks were enriched in 5'UTR of the CTNNB1 gene from m^{6}A RIP-seq data. Squares mark m^{6}A peaks in HeLa cells;

(B) m^{6}A RIP-qPCR analysis of CTNNB1 mRNA in HeLa and HEK293T cells using m^{6}A antibody;

(C) mRNA levels of CTNNB1 in HEK293T cells transfected with wild-type Cas13b and NT-gRNA or individual gRNAs for 24 h;

(D) Threshold cycle (Ct) of qPCR showing SELECT results for detecting the m^{6}A S3 site in CTNNB1 in HEK293T cells transfected with NT-gRNA (control) or gRNA1/2/3,

respectively, for 24 h, with fold change listed;

(E) Threshold cycle (Ct) of qPCR showing SELECT results for detecting the m^6A S1 site in CTNNB1 in HEK293T cells transfected with dCas13b-ALKBH5 H204A combined with NT-gRNA (control) or gRNA1/2/3, respectively, for 24 h, with fold change listed;

(F) Threshold cycle (Ct) of qPCR showing SELECT results for detecting the m^6A S2 site in CTNNB1 in HEK293T cells transfected with dCas13b-ALKBH5 H204A combined with NT-gRNA (control) or gRNA1/2/3, respectively, for 24 h, with fold change listed;

(G) mRNA expression of CTNNB1 in HEK293T cells transfected with dCas13b-ALKBH5 H204A combined with NT-gRNA or gRNA1/2/3, respectively, for 24 h;

(H) Representative scheme of the reporter assay: 5'UTR of CTNNB1 was inserted prior to the F-Luc region of pGL-Basic plasmid.

Data are presented as mean ± SD from three independent experiments. ** $p < 0.01$ by Student's *t* test (B) or One-Way ANOVA (C).

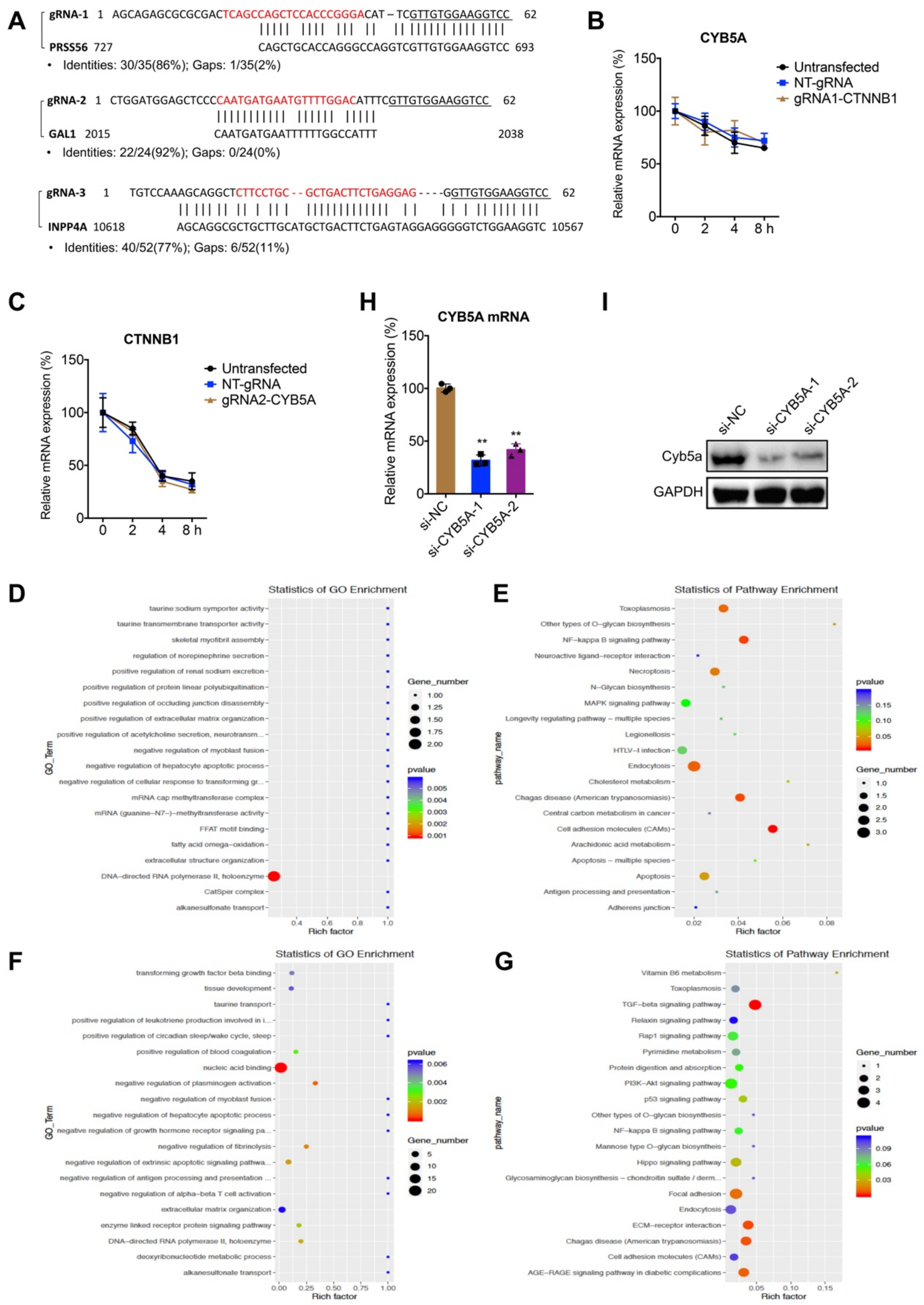

Figure S4. Specificity of dm^{6}ACRISPR on m^{6}A demethylation.

(A) Sequence alignment between CYB5A gRNAs and PRSS56, GALR1 or INPP4A mRNAs. The central regions of gRNAs are marked in red, and gRNA loop regions (partial) are

underlined;

(B) HEK293T cells were transfected with dCas13b-ALKBH5 combined with NT-gRNA or gRNA1-CTNNB1 (gRNA-1 targeting CTNNB1), respectively, for 24 h, followed by treatment of Act-D at the indicated time period. Expression levels of CYB5A mRNA were measured by RT-qPCR;

(C) HEK293T cells were transfected with dCas13b-ALKBH5 combined with NT-gRNA or gRNA2-CYB5A (gRNA-2 targeting CYB5A), respectively, for 24 h, followed by treatment of Act-D at the indicated time period. Expression levels of CRNNB1 mRNA were measured by RT-qPCR;

(D&E) Cluster profiler identified the gene ontology (GO, D) or KEGG (E) processes of 154 m^6A peaks with a significant change ($p < 0.05$) in gRNA-1 for the CYB5A group when compared with that in the NT-gRNA group;

(F&G) Cluster profiler identified the gene ontology (GO, F) or KEGG (G) processes of 99 mRNA with a significant change ($p < 0.05$) in gRNA-1 for the CYB5A group when compared with that in the NT-gRNA group;

(H&I) Cells were transfected with siRNA negative control (si-NC) or siRNAs for CYB5A for 24 h, with mRNA (H) or protein (I) expression of CYB5A measured;

Data are presented as mean ± SD from three independent experiments. ** $p < 0.01$ by One-Way ANOVA.

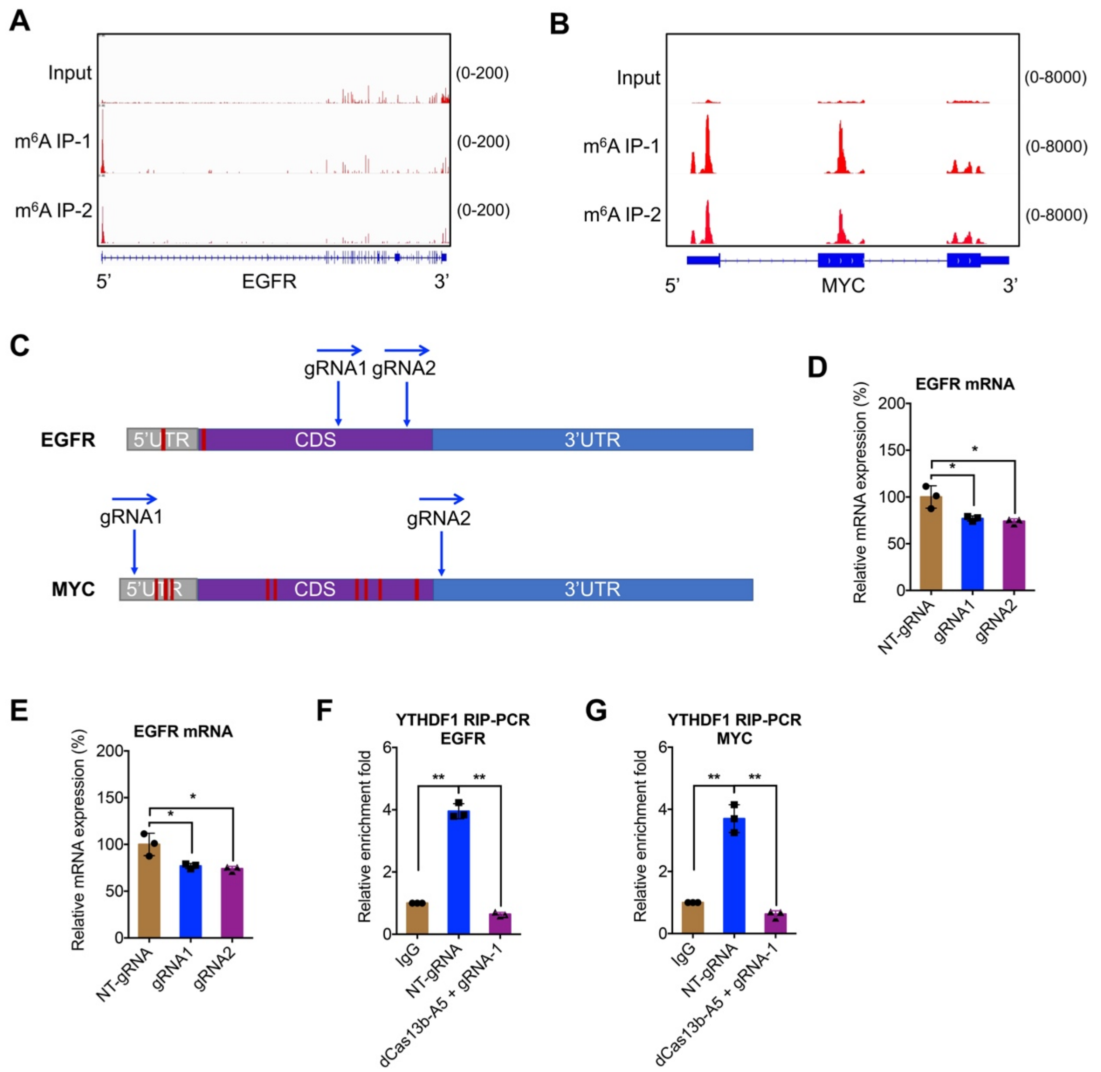

Figure S5. Targeting m^{6}A of oncogene transcripts by dm^{6}ACRISPR regulates cell proliferation.

(A&B) m^{6}A peaks in EGFR (A) and MYC (B) genes from m^{6}A RIP-seq data;

(C) Schematic representation of positions of m^{6}A sites within EGFR or MYC mRNA and regions targeted by individual gRNAs;

(D&E) mRNA levels of EGFR (D) or MYC (E) in HeLa cells transfected with dCas13b-ALKBH5 combined with NT-gRNA or gRNA1/2, respectively, for 24 h;

(F&G) YTHDF1 RIP-qPCR in HeLa cells transfected with dCas13b-ALKBH5 combined with NT-gRNA or gRNA for EGFR (F) or MYC (G) for 24 h.

Data are presented as mean ± SD from three independent experiments. *$p < 0.05$, ** $p < 0.01$ by One-Way ANOVA with Bonferroni test.

Table S1. Sequences of gRNAs used in this study

Name	Guide RNA sequence (5'-3')
gRNAcyb5a-1	AGCAGAGCGCGCGACTCAGCCAGCTCCACCCGGGACATTC GTTGTGGAAGGTCCAGTTTTGAGGGGCTATTACAAC
gRNAcyb5a-2	CTGGATGGAGCTCCCCAATGATGAATGTTTTGGACATTTCGT TGTGGAAGGTCCAGTTTTGAGGGGCTATTACAAC
gRNAcyb5a-3	TGTCCAAAGCAGGCTCTTCCTGCGCTGACTTCTGAGGAGG GTTGTGGAAGGTCCAGTTTTGAGGGGCTATTACAAC
gRNActnnb1-1	GGGGCCGGGCCAACGCTGCTGCCACAGACCGAGAGGCTTG TTGTGGAAGGTCCAGTTTTGAGGGGCTATTACAAC
gRNActnnb1-2	ATAGGTCCTCATTATATTTACTAAAGCTTGGGGTCCACCAGT TGTGGAAGGTCCAGTTTTGAGGGGCTATTACAAC
gRNActnnb1-3	CTTCTTGAGTCACTCCCAAAATCCATTTGTATTGTTACTCGT TGTGGAAGGTCCAGTTTTGAGGGGCTATTACAAC
gRNAfluc-1	GTAATCCTGAAGGCTCCTCAGAAACAGCTCTTCTTCAAATG TTGTGGAAGGTCCAGTTTTGAGGGGCTATTACAAC
gRNAfluc-2	TTATCATGTCTGCTCGAAGCGGCCGGCCGCCCCGACTCTAG TTGTGGAAGGTCCAGTTTTGAGGGGCTATTACAAC
gRNAegfr-1	AGGCACTGGGAGGAAGGTGTCGTCTATGCTGTCCTCAGTCG TTGTGGAAGGTCCAGTTTTGAGGGGCTATTACAAC
gRNAegfr-2	GGATGGAGGAGATCTCGCTGGCAGGGATTCCGTCATATGGG TTGTGGAAGGTCCAGTTTTGAGGGGCTATTACAAC
gRNAmyc-1	TTCCCAGGACGCCCGCAGCGCAGCTCTGCTCGCCCGGCTC GTTGTGGAAGGTCCAGTTTTGAGGGGCTATTACAAC
gRNAmyc-2	ATAGGTGATTGCTCAGGACATTTCTGTTAGAAGGAATCGT G TTGTGGAAGGTCCAGTTTTGAGGGGCTATTACAAC

Table S2. Primers for SELECT qPCR

Name	Sequence (5'-3')
CYB5A-X-up	TAGCCAGTACCGTAGTGCGTGGTGGTTGTGCTTCTGAATC
CYB5A-X-down	5PHOS/CCTCTAGGGTGTAGTACTTCCAGAGGCTGAGTCG CTGCAT
CYB5A-N-up	TAGCCAGTACCGTAGTGCGTGTTCTGAATCTCCTCTAGGG
CYB5A-N-down	5PHOS/GTAGTACTTCACGGCCTCGTCAGAGGCTGAGTCG CTGCAT
CTNNB1-X1-up	TAGCCAGTACCGTAGTGCGTG CAGCTTGAGTAGCCATTG
CTNNB1-X1-down	5PHOS/CCACGCTGGATTTTCAAAACAGAGGCTGAGTCGC TGCAT
CTNNB1-X2-up	TAGCCAGTACCGTAGTGCGTG TCCTCCCGCCGCGGGAG
CTNNB1-X2-down	5PHOS/CCGACCGTCCTCGACCTGCCAGAGGCTGAGTCGC TGCAT
CTNNB1-X3-up	TAGCCAGTACCGTAGTGCGTG CAGCTTGAGTAGCCATTG
CTNNB1-X3-down	5PHOS/CCACGCTGGATTTTCAAAACAGAGGCTGAGTCGC TGCAT
CTNNB1-N1-up	TAGCCAGTACCGTAGTGCGTGGAGTCCGACCGTCCTCGA CC
CTNNB1-N1-down	5PHOS/GCGGTGGCGGCTCGCAGAGGCTGAGTCGCTGCAT
CTNNB1-N3-up	TAGCCAGTACCGTAGTGCGTGAGTAGCCATTGTCCACGC
CTNNB1-N3-down	5PHOS/GGATTTTCAAAACAGTTGTATGGCAGAGGCTGAG TCGCTGCAT
CTNNB1FLUC-X3-up	TAGCCAGTACCGTAGTGCGTGGTTTTTGGCGTCTTCCATT G
CTNNB1FLUC-X3-down	TAGCCAGTACCGTAGTGCGTGGTCTTCCATTGTCCACGC
qPCR-SELECT-Forward	ATGCAGCGACTCAGCCTCTG
qPCR-SELECT-Reverse	TAGCCAGTACCGTAGTGCGTG

Table S3. Primers for PCR

Gene	Forward (5'-3')	Reverse (5'-3')
Plasmid mutation		
Cas13b-A133H	CAGGGACCTGACCAACCACTACAAGACCTACGA	TCGTAGGTCTTGTAGTGGTTGGTCAGGTCCCTG
Cas13b-A1058H	CCGGAACGCCTTCGATCACAACAATTACCCCGA	TCGGGGTAATTGTTGTGATCGAAGGCGTTCCGG
qRT-PCR		
GAPDH	GTCTCCTCTGACTTCAACAGCG	ACCACCCTGTTGCTGTAGCCAA
CYB5A	GTTTTAAGGGAACAAGCTGGAG	TCCACCAACTGGAACTAGAATC
CTNNB1	TGGATTGATTCGAAATCTTGCC	GAACAAGCAACTGAACTAGTCG
FLUC	TGGAAGATGGAACCGCTGGAGAG	TTCATAGCTTCTGCCAACCGAACG
PRSS56	GCTGTGCTGCTGCTGCTACC	CTGCCTGCAACGCCTGAGTC
GALR1	TTCCTTCCTCTTCAGAATCACC	CGAATGTGACACTTGAACACTT
INPP4A	GAATCCGATCCAAATACGCTTC	CTCTAGTAGGAGCTTCACGAAC
ZNF581	GCATTTTCCTCCGTTGAGACC	CCCTGAGTGTCAATAAGCAGGTA
EXOSC2	TGGCTCGCAAGCCTCTTAG	TGTGTCCGTAGTGATTGTATCCC
PPAN	GGCAAAGTGATGTTCCACAGT	TCGCCGTCCTTGAAGGGAT
MAPRE2	GCCTATTGCCAATTCATGGACA	TCGCTTAAATGATGCTTGCAGA
EGFR	ACCCATATGTACCATCGATGTC	GAATTCGATGATCAACTCACGG
MYC	CAGCTGCTTAGACGCTGGATT	GTAGAAATACGGCTGCACCGA

Materials and Methods

Total RNA isolation and quantitative PCR

After transfection with Cas13b/PspCas13b fusion and gRNA plasmids for 24 h, total RNAs were harvested and extracted by TRIZOL (Invitrogen) according to the protocol used in our previous study (1). Extracted total RNAs were quantified and reverse-transcribed into cDNA using PrimeScript RT Reagent Kit (TaKaRa). All qPCR reactions were performed in 10-μl reactions using TB Green™ Premix Ex Taq™ II (TaKaRa). Primers for qPCR are listed in Table S3. GAPDH were used as the endogenous control and target C_t values were normalized to non-targeting (NT) gRNA C_t value. Relative abundance was determined using $2^{-\Delta Ct}$. All assays were performed with three independent biological replicates.

Immunofluorescent assay

HEK293T cells were seeded onto coverslips in a 4-well plate at a density of 1×10^4 cells per well. After transfection for 24 h, cells were rinsed with PBS and fixed in 4% paraformaldehyde. The cells were permeabilized with 0.1% Triton X-100 in PBS and blocked with 0.1% Tween 20 and 1% horse serum in PBS. Cells were incubated with anti-ALKBH5 antibody (ABE547, Millipore), followed by FITC-labeled Goat Anti-Rabbit IgG (H+L) (A0562, Beyotime). Cell nuclei were stained with Hochest. Localization of ALKBH5 was imaged using Zeiss LSM710 fluorescence microscope.

Western blot analysis

Protein levels were measured by Western blot analysis according to our previous study (2). In brief, after transfection for 24 h, cells were collected and mixed with 1x SDS loading dye (50 mM Tris-HCl, pH 6.8, 2% SDS, 10% glycerol, 1% β-mercaptoethanol, 0.1% bromophenol blue) and then incubated at 95°C for 15 min. Samples were separated by SDS-PAGE and transferred to PVDF membrane. Primary antibodies used for immunoblotting include anti-Cyb5a (ab69801, Abcam), anti-β-catenin (#8480S, Cell Signaling Technology), anti-ALKBH5 (ABE547, Millipore), anti-EGFR (ab272293, Abcam), anti-Myc (ab39688, Abcam), and anti-GAPDH (AP0063, Bioworld). Immunoblotting results presented are representative from at least three independent experiments.

Cell proliferation

Cell proliferation was tested by using the CCK-8 kit (Dojindo, Gaithersburg, MD) according to our previous study (3,4).

Sub-cellular fractionation

After transfection with dCas13b-ALKBH5 or ALKBH5-dCas13b for 24 h, HEK293T cells were rinsed with PBS once. Fractionation of nuclear and cytoplasmic fractions was performed using the NE-PER(R) nuclear and cytoplasmic extraction kit (Thermo Fisher). Nuclear and cytoplasmic fractions, a total of 60 μg proteins each, were loaded for Western blot analysis.

References

1. Chen, Z., Qi, M., Shen, B., Luo, G., Wu, Y., Li, J., Lu, Z., Zheng, Z., Dai, Q. and Wang, H. (2019) Transfer RNA demethylase ALKBH3 promotes cancer progression via induction of tRNA-derived small RNAs. *Nucleic Acids Res*, **47**, 2533-2545.
2. Lu, L., Chen, Z., Lin, X., Tian, L., Su, Q., An, P., Li, W., Wu, Y., Du, J., Shan, H. *et al.* (2020) Inhibition of BRD4 suppresses the malignancy of breast cancer cells via regulation of Snail. *Cell Death Differ*, **27**, 255-268.
3. Zhou, Y., Lu, L., Jiang, G., Chen, Z., Li, J., An, P., Chen, L., Du, J. and Wang, H. (2019) Targeting CDK7 increases the stability of Snail to promote the dissemination of colorectal cancer. *Cell Death Differ*, **26**, 1442-1452.
4. Lin, X., Chai, G., Wu, Y., Li, J., Chen, F., Liu, J., Luo, G., Tauler, J., Du, J., Lin, S. *et al.* (2019) RNA m(6)A methylation regulates the epithelial mesenchymal transition of cancer cells and translation of Snail. *Nat Commun*, **10**, 2065.
